# Supplementary material for: Are carbon emissions trading and green financial instruments synergistic? -Comprehensive quantitative research based on content analysis
Source: PLoS One. 2024 Mar 7;19(3):e0298601. doi: 10.1371/journal.pone.0298601 (PMC10919601; doi:10.1371/journal.pone.0298601)
Supplement: S1 Data — (ZIP) [file pone.0298601.s001.zip › Data/地级市Z_F工作报告.zip!/╘╞─╧/└Ñ├≈╩╨/└┤╫╘╣1⁄2╓┌║┼í╛┬φ┐╦╩2╛▌═°í┐_2018.docx]

市 长 王喜良

各位代表：

我代表市人民政府，向大会报告工作，请予审议，并请各位政协委员提出意见。

一、 2017年工作回顾

过去一年，在省委、省政府和市委的坚强领导下，在市人大及其常委会的法律监督和工作监督下，在市政协的民主监督支持下，我们深入学习宣传贯彻习近平新时代中国特色社会主义思想和党的十九大精神，全面贯彻落实习近平总书记对云南发展的重要指示精神，坚持稳中求进工作总基调，深入践行新发展理念，按照当好全省经济社会发展排头兵的要求，认真落实市第十一次党代会、市委十一届二次全会的安排部署，齐心协力、攻坚克难，较好地完成了市十四届人大一次会议确定的目标任务。全市经济社会呈现稳中向好的良好态势，质量效益有了新提升，发展动能有了新亮点，经济活力有了新增强，生态环境有了新改善，民生事业有了新进展。全市实现地区生产总值4857.6亿元，增长9.7%；一般公共预算收入560.9亿元，增长8.2%；固定资产投资4217.9亿元，增长7.6%；社会消费品零售总额2591亿元，增长12.2%；城镇和农村常住居民人均可支配收入分别达39788元、13698元，分别增长8.3%、9.1%；居民消费价格上涨0.5%；城镇登记失业率为3%。

一年来，我们主要做了以下工作。

（一）凝心聚力抓落实，区域性国际中心城市建设迈出坚实步伐

举全市之力，聚各方之智，抓好区域性国际中心城市实施纲要的组织实施，“一枢纽”“四中心”“三品牌”建设取得新进展。

“一个枢纽”建设步伐加快。国家综合交通枢纽示范城市建设取得新突破，晋红、宜澄、易武等高速公路建成通车，昆明与滇中城市的互联互通水平进一步提升。昆明站至昆明南站高铁、昆明至成都、重庆动车开通，沪昆、云桂高铁带动效应显现。国际通信枢纽和信息汇集中心建设全面提速。《昆明国际航空枢纽战略规划》印发实施，长水机场列为国际航空枢纽机场规划建设。开通中欧、中亚等多式联运国际货运班列，新开通昆明至莫斯科等48条国内外客运航线，开通昆明至班加罗尔等7条全货机航线，长水机场旅客吞吐量4473万人次，增长6.6%，昆明与世界的联系更加紧密。

“四个中心”辐射能力得到加强。经济贸易中心建设取得新突破，成功举办商洽会、国际旅交会等国际经贸展会，综合保税区一期通过国家验收并投入运行，高新保税物流中心正式运营，腾俊国际陆港保税物流中心通过国家验收，全年进出口总额78.2亿美元、增长18.2%，扭转了连续两年负增长的不利局面。科技创新中心建设取得新成效，面向南亚东南亚的知识产权服务中心落户昆明。出台国际金融服务中心建设实施方案、国际金融小镇建设实施方案及扶持奖励办法，国际金融小镇建设正式启动，引进大华银行、平安证券、锦泰保险等16家金融机构。人文交流中心建设迈出新步伐，与捷克奥洛莫茨市结为国际友城，与法国南锡市结为友好交流城市，昆明国际友城旅游联盟正式成立。上合组织青年交流中心在昆明学院正式挂牌，上合国际马拉松、世界龙舟赛等人文交流日益频繁，成功举办中国昆明郑和国际文化旅游节，在昆留学生人数突破1万人，接待海外旅游者134万人次。

“三大品牌”效应逐步显现。全力实施创建全国文明城市“五大工程”，对标开展“九大环境”整治，建立常态长效创建机制，市民文明素质、城市文明程度、群众满意度明显提升，安宁成功创建为全国文明城市。斗南花卉小镇入围全国一流特色小镇，全市鲜切花产量达55.4亿枝，远销46个国家和地区，斗南花卉市场作为亚洲最大鲜切花交易市场的地位更加巩固，春城花都的品牌进一步擦亮。成立历史文化名城保护委员会和专家委员会，加快推进历史文化城市品牌建设工作。翠湖周边历史文化片区整治提升工程稳步实施，“两坡一街一河”改造提升工程初见成效。云南陆军讲武堂提升改造稳步推进，启动西南联大周边历史文化片区保护和景观提升规划工作，文庙恢复性修建一期工程主体完工，历史文化名城吸引力不断提升。大健康发展规划、健康服务业发展规划发布实施，中国昆明国家大健康产业示范区、国家植物博物馆建设项目获得国家支持，示范区核心区规划布局和实施方案加快编制，滇池国际养生养老度假区建成运营，绿地健康城、云白药健康产业园等项目加快推进，华大基因、康美药业、鹏瑞利健康城等16个国内外健康类项目签约落地，协议总投资约1500亿元，“中国健康之城”影响力逐步提升。

（二）全力以赴稳增长，经济发展质量和效益不断提升

把稳增长作为首要任务，精准施策破难题，降本增效稳工业，多措并举育新兴，经济增速好于预期、高于同期，发展质量明显提升。全市地区生产总值增速高于上年1.2个百分点，为昆明近4年来的新高；税收收入完成410.9亿元，增长12.4%，规模以上工业企业利润增长65.7%。

稳增长政策效应显现。年初制定经济工作开门红实施方案，出台26条稳增长政策措施，市级领导带头深入企业、深入基层开展稳增长督查，推动全市经济运行稳中向好。安排6935万元市级资金，支持493户企业扩销促产，兑现49户企业物流补贴1418万元，企业停减产面较上年最高值下降10个百分点。加大市级领导结对帮扶工作力度，帮助182户工业企业解决生产经营困难问题370多个。安排8.87亿元工业发展引导资金，以股权+债权方式，有效缓解了149户企业流动资金周转困难。认真落实电力市场化交易改革措施，降低工业用电成本超过19亿元。工业经济攻坚战初战告捷，全市规模以上工业增加值增长10.4%，创2014年以来新高。着力解决“烂尾楼”，问题导向、因情施策，采取合理调整商住比例等有效措施，激活“僵尸企业”“僵尸项目”，房地产投资增长10%，稳住了全市经济增长的基本盘。坚持把发展现代服务业作为经济转型突破口，着力打好服务业攻坚战，服务业进一步提质增效，增速较上年提高1.3个百分点。

新动能培育呈现新亮点。深圳优必选机器人实现量产，中石油炼油项目及云天化配套异辛烷、聚丙烯项目投产，中铁电建大型盾构机、富信智能终端等42个亿元以上项目竣工，新增新建投产企业22户、小升规企业68户，新增工业产值560亿元左右。凝慧氮化镓、中国医学科学院疫苗产业基地、中汽中心、东风云汽等49个亿元以上项目开工，紫光芯云产业园、协鑫集团30GW单晶硅、航天神州新能源汽车、宝能集团系列项目等一批重大项目落户昆明，汉能移动能源产业园、通盈集团“双环铂”、江铃新能源汽车等23个重大项目签约，计划总投资1400亿元。全市电子信息、生物医药制造业增加值增速分别达22.6%和17.5%，分别高于全部工业增加值增速12.5和7.4个百分点。

新业态新模式新经济蓬勃发展。主动适应经济发展新常态，秉持开放包容理念，着力推动新技术、新业态、新模式等新经济快速发展。总部（楼宇）经济培育取得明显成效，中交建南亚总部、中铁建西南总部、保利大厦、景成大厦四个项目在巫家坝中央商务区正式开工。新增华侨城、中铁等24户总部企业，税收超过千万元楼宇达31幢、亿元以上楼宇达19幢。文化建设和产业发展“510”工程深入实施，建成文创产业园区21个，昆明入选国家文化消费试点城市。重拳整治旅游市场秩序，全力推动旅游业转型升级，古滇名城温泉山庄等13个重点旅游项目建成运营，万达文化旅游城、蓝光欢乐城等项目开工建设，华强方特落地昆明，全市接待游客突破1.3亿人次、增长31.9%，旅游总收入1608.7亿元、增长49.8%。会展经济发展势头良好，举办农博会、医博会等展会120场，会展业总收入超过26.1亿元，增长18.6%，被评为2017最具竞争力会展城市。网络订餐、共享汽车、共享单车等新业态快速发展。新兴农业经营主体不断壮大，新增市级农业龙头企业68家，都市农庄8座，农村三次产业融合发展步伐加快。“互联网+农业”快速发展，东川、寻甸入围国家级电子商务进农村综合示范县。

新增长点新增长极不断培育壮大。呈贡信息产业园建设步伐加快，浪潮云计算产业园一期投入运营，中国移动云南数据中心、启明星辰信息安全产业园等项目开工建设，华为、微软、谷歌等400户企业入住，总投资达200亿元。健全与滇中新区规划、资源和重大项目推进等融合联动机制，在打基础、育产业、强招商、优环境等方面取得丰硕成果。高新区、经开区、度假区提速发展，高新区营业收入突破2000亿元，经开区综合发展水平在全国排位升至第40位，全市经济增长新引擎的基础不断夯实。

（三）加强城乡规划建设管理，人居环境品质明显提升

坚持以规划为引领，以“五网”建设为重点，以智慧城市建设为抓手，城市品质不断提升。

规划引领不断加强。全年召开规委会18次，充分发挥了规划的龙头牵引作用。昆明被列为全国城市总体规划和土地利用总体规划编制试点城市。坚持开放思维、国际视野，委托国内外一流团队编制了昆明2050城市发展战略规划、昆明—滇中新区远景空间发展规划、昆明城市建筑特色导引等一系列重大规划，科学谋划城市发展新格局，全力塑造昆明未来新形象。“多规合一”信息平台开通运行，城乡规划条例修订颁布，城乡规划的系统化、科学化和法制化水平进一步提升。

城乡建设水平得到提升。完成27条城市道路建设和15项道路整治。人民路道路恢复提升工程开工建设，完成东风路恢复提升工程、二环石虎关立交至大观河桥段高架桥提升改造工程前期工作。统筹市政、人防、轨道工程建设，编制金马坊节点改造工程规划方案。开工建设春雨路、古滇路地下综合管廊，建成地下综合管廊10.2公里。编制实施海绵城市建设专项规划，启动海绵城市建设25平方公里。草海、巫家坝、东白沙河、滇池国际会展中心等重点片区开发建设加快推进。新增城镇户籍人口12万人，户籍人口城镇化率达59.5%。建成省级美丽宜居乡村85个，新农村建设扎实推进。

“五网”建设成效明显。功东、福宜等12条高速项目建设加快推进，新昆嵩等4条高速公路建成通车，新增高速公路通车里程119公里。昆玉高速鸣泉收费站完成外迁，主城至马金铺段实现免费通行。星耀立交建成使用，罗衙、马郎立交开工建设。完成通村公路路面硬化830公里，路基改造550公里，为脱贫攻坚创造了良好的交通条件。地铁3号线、6号线一期投入试运营，与1、2号线首期形成大“十”字骨架线网，日均客流量达47万人次，市民出行更加便捷。长水机场航站区改扩建工程开工，昆明被列为国家通用航空产业综合示范区。新增城市宽带接入网络100万户，开通室外无线宽带接入网络站点6150个。主城区天然气置换全面完成，新建新能源汽车充电桩1887个、充电站41个。建成海马箐、真金万中型水库，滇中引水骨干工程控制性工程开工建设、昆明受水区前期工作全面启动，柴石滩大型灌区工程顺利推进，圆满完成年度中小型水库、河道整治及3.3万件五小水利工程建设任务，城乡供水保障能力进一步提高。

精细化管理水平不断提升。启动智慧城市项目建设，智慧城管“五进网格”工作机制有效建立，市、区、街道三级智慧城管系统联通运行。拆除违法违规建筑1108万平方米，完成82个“三旧”项目改造。“厕所革命”全面推开，新建和改造提升城市公厕612座。五华、盘龙、官渡、西山、经开区等实现环卫一体化。乡镇生活垃圾处理设施、自来水供水设施、公厕覆盖率达100%。出台缓解交通拥堵16条措施，新开行公交线路35条，新增停车场32个、停车泊位2.6万个，规范发展公共自行车、共享单车，市民出行方式更加灵活。

（四）全力推动改革创新，发展动力活力不断增强

坚持以改革破解难题，以创新引领发展，发展动力和创造活力不断释放。

重点领域改革成效明显。供给侧结构性改革取得新进展，化解煤炭行业过剩产能24万吨，提前完成“十三五”煤炭去产能任务。房地产市场平稳健康发展，商品房去化周期显著缩短。政府债务置换377.7亿元，45个政府投资项目通过PPP模式筹措资金1300亿元。认真落实减税降费政策，减轻企业负担353.8亿元。“放管服”改革深入推进，出台“最多跑一次”改革、建设“四个一”国际营商环境等实施意见，“一网四中心”建设加快推进，公布102项“最多跑一次”事项清单，取消13项证明材料。23个部门50项涉企证照事项全部整合到营业执照上，企业登记实现“多证合一”和全程电子化。在具有行政审批权的市级部门设立“行政审批处”，在主城区和开发（度假）园区组建“行政审批局”，公共资源交易电子化实现全国首例跨省远程异地评标，营商环境持续改善。宜良成为全国农村集体产权制度改革试点县，理顺倘甸轿子山两区管理体制。投融资、教育、文化、医疗、社保等领域改革有序推进。

创新活力不断激发。深入实施创新驱动发展战略，科技创新平台倍增计划和科技服务业发展倍增计划稳步推进，经开区入选国家“双创”示范基地。成功举办金砖国家创客大赛、“春城创业荟”创业创新大赛等活动。新增省级创业园区、众创空间16个。与22名国家“千人计划”专家签订服务昆明协议。中科院天元数学国际交流平台项目落户昆明。新增市级重点实验室9个、工程技术研究中心15个、院士工作站6个。科技服务业营业收入增长14%。民营经济发展态势良好，新增民营企业1.8万户，增长8%。

产业招商成效显著。坚持大招商、招大商，积极开展以商招商、中介招商、上门招商，设立15个重点产业招商分局，实行重大项目市级领导经理负责制，招商引资精准度明显提高，项目签约率、落地率、开工率明显提升，引进市外到位资金1043.7亿元、增长14%，实际利用外资8.01亿美元、增长8.3%。新引进美国微软、德国费森尤斯等世界500强6家，中铁建、中核等中国500强6家，深圳华强、上海大华2家中国民营企业500强企业，与东胜集团、财信集团、东方园林、首创等170户行业龙头企业签约，投资额达4350亿元，进一步夯实了我市产业发展的项目支撑。

（五）狠抓滇池治理，生态环境建设成效明显

牢固树立和践行绿水青山就是金山银山的理念，生态建设和环境保护力度进一步加大，绿色发展取得新进步。

滇池治理取得新突破。全面深化河长制，建立“四级河长五级治理体系”，设立河长3489名，实现河道、管网清淤常态化，滇池污染存量不断削减。海洪、大湾、西华等6个湿地项目加快建设，湿地净化水质效用日益显现。开展水质净化厂出水水质提标工作，完成了第一、第九及古城水质净化厂超极限除磷试验示范项目建设。全面推行滇池流域河道生态补偿，生态补偿金达4.86亿元。实施滇池保护治理项目100个，完成投资36.3亿元，25条入湖河道水质达标，滇池全湖水质稳定保持在Ⅴ类。

生态环境保护取得新成效。认真做好中央、省环保督查反馈意见整改，做到件件落实。大力开展滇池流域及西山重点区域“五采区”整治，关停采矿采砂采石点72个。退耕还林、人工造林、园林绿化等工程有力推进，完成营造林57万亩，新增城市绿地360.8公顷。石林成为国家生态文明示范县，富民、宜良创建为国家园林县城。万元GDP能耗预计下降3%以上，万元GDP用水量比2015年下降10.6%，圆满完成省下达的污染物减排目标任务，经开区入选国家第一批绿色园区示范名单，云内动力等4户企业入选国家第一批绿色工厂示范名单。全市环境空气质量总体优良，达到国家二级标准。

（六）全力打好脱贫攻坚战，群众福祉稳步提升

坚持民生为要，“十件惠民实事”全面完成，民生支出占一般公共预算支出75.2%。

脱贫攻坚推进有力。围绕“两不愁，三保障”目标，落实“两出两进两对接一提升”工作举措，实现贫困对象动态管理、分类施策、精准帮扶，突出抓好产业就业促进增收，全面推进义务教育、基本医疗、住房安全、饮水安全有保障。筹措各类扶贫资金113.8亿元，推进贫困村“菜单式”产业扶贫，消除348个集体经济“空壳村”，完成农村危房改造12.5万户，建档立卡户易地扶贫搬迁累计入住1162户4317人，贫困人口转移就业3.9万人，8.1万贫困人口脱贫，128个贫困村出列，寻甸县已申报国家贫困县退出评估验收，禄劝县达到预期标准。

社会保障水平稳步提高。城镇新增就业14.6万人，实名登记高校毕业生就业率达94.7%。开展城乡劳动力职业技能培训5.8万人，完成农村劳动力转移就业18.1万人，实现转移收入23.4亿元。各项社会保险参保新增28.5万人，参保覆盖率保持在96%以上。建成城镇保障性安居工程3万套，解决13万人的住房困难问题。落实社会救助制度，18.4万人纳入城乡低保。多层次养老服务体系初步建立，建成社区居家养老服务中心36个、民办养老机构10个，新增养老床位4780张。

社会事业加快发展。坚持教育优先战略，在全省率先实现义务教育基本均衡。“三名”工程深入推进，清华附中、黄冈中学等国内14所名校到昆办学，引进名校长13名、名师59名，优质教育资源规模不断扩大。昆明学院获批硕士学位授予单位。云南阜外心血管病医院投入使用，北京安贞医院昆明技术合作医院成立。市妇幼保健院成为国家级儿童早期发展示范基地，市第一人民医院、延安医院获批国家干细胞临床研究备案机构。孕产妇死亡率、婴儿死亡率分别下降到十万分之13.48和千分之3.73。全市乡镇卫生院全部达标，昆明成为国家幸福家庭示范城市。建成200个“15分钟体育健身圈”项目，成功举办第五届中国聂耳音乐（合唱）周、第十届中国金唱片奖系列活动，荣获中国十大最具文化影响力城市称号。

社会保持和谐安定。矛盾纠纷排查化解取得新成效，落实党政领导包案化解信访积案责任制，信访量持续下降。组织开展“风雷行动”攻坚战，严厉打击“两抢一盗”、黄赌毒、黑拐骗以及电信网络诈骗等违法犯罪行为，刑事案件下降22.2%，社会大局持续稳定。成功举办市第十一届少数民族传统体育运动会，民族团结进步示范创建活动深入开展，石林成为全国民族团结进步创建示范区。“军人之家”建设全面推进，军政军民团结良好局面不断巩固。全面抓好安全生产、食品药品安全、道路交通安全等工作，安全生产形势总体平稳。

各位代表！一年来，我们主动接受市人大及其常委会的法律监督和工作监督，自觉接受市政协的民主监督，积极接受社会监督，办理人大代表建议440件、政协委员提案462件。制定政府规章5件，提请市人大常委会审议地方性法规12件。我们认真贯彻落实中央八项规定精神，深入推进“两学一做”学习教育常态化制度化，坚决落实中央巡视“回头看”整改要求，严格落实党风廉政建设责任制，强化行政监察和审计监督，严肃查处一批不正之风和腐败行为。我们发扬“跨越发展、争创一流；比学赶超、奋勇争先”的精神，持续开展“政府提效率，企业增效益”活动，坚决整治“不作为、乱作为、慢作为”，着力营造干事创业良好氛围，干部作风明显改善。国家安全、防灾减灾、港澳台侨、民族宗教、档案史志等工作取得新成绩。

各位代表！过去一年取得的成绩，是党中央国务院亲切关怀的结果，是省委省政府和市委坚强领导的结果，是全市干部群众团结奋斗的结果。在此，我代表市人民政府，向全市各族人民，向市人大代表和政协委员，向各民主党派、工商联、无党派人士、各人民团体和社会各界人士，向中央、省驻昆单位和省级各部门，向驻昆解放军和武警部队官兵，向所有关心支持昆明发展的各界友人，表示衷心的感谢和崇高的敬意！

总结回顾过去的工作，做好政府工作的基本经验和根本遵循是：必须坚持党的领导，坚决维护党中央权威和集中统一领导，牢牢把握正确的政治方向，不折不扣落实省委、省政府和市委的决策部署，奋力开创区域性国际中心城市建设新局面；必须坚持主动服务和融入国家发展战略，抢抓机遇，深化改革，扩大开放，在更宽视野、更高层次、更大格局中谋求发展，努力当好全省经济社会发展排头兵；必须坚持把发展作为第一要务，主动适应把握引领经济发展新常态，不断深化供给侧结构性改革，培育发展新动能，构建现代化经济体系，推动经济高质量发展，不断增强城市综合实力和竞争力；必须坚持以人民为中心，把提高保障和改善民生水平作为政府一切工作的出发点和落脚点，不忘初心，牢记使命，不断增强群众的获得感幸福感安全感，汇聚推动昆明跨越发展的磅礴力量。

我们清醒地认识到，建设区域性国际中心城市还有许多短板和弱项，工作中仍然存在一些差距和不足，主要表现在：一是发展不平衡不充分的矛盾突出。经济总量不大、发展速度不快，工业不强、服务业不优、民营经济活力不足，城乡区域发展不平衡等问题比较突出，脱贫攻坚任务艰巨。二是离“跨越发展、争创一流；比学赶超、奋勇争先”的工作要求还存在较大差距。标兵越来越远，追兵越来越近，与先进发达省会城市的差距还在扩大。三是离人民对美好生活的需要还有较大差距。群众在教育、医疗、养老、住房等方面的操心事、烦心事还不少。四是落实“马上办、真抓实干”的工作作风有明显差距。一些政府工作人员能力素质不适应，遇事“拖、躲、绕、滑”，存在能力不足不会为、动力不足不想为、担当不足不敢为的现象。五是营商环境还不够优，“玻璃门”“弹簧门”“旋转门”依然存在。主动服务、上门服务做得还不够好，企业和群众还有不少的差评，没有完全实现“最多跑一次”。六是严格履行党风廉政建设主体责任和“一岗双责”还不够到位。“四风”顽疾尚未根除，形式主义、官僚主义在一定程度上仍然存在。对这些问题，我们要高度重视，切实采取有力措施，认真加以解决。

二、 2018年工作安排

今年，是全面贯彻落实党的十九大精神的开局之年，是改革开放40周年，是实施“十三五”规划承上启下的关键一年，也是我市在全省率先全面建成小康社会的决胜之年。我们要全面贯彻党的十九大精神，以习近平新时代中国特色社会主义思想为指导，进一步贯彻落实习近平总书记对云南发展的重要指示精神，认真落实省委十届四次全会、省“两会”和市委十一届四次全会的决策部署，坚持稳中求进工作总基调，践行新发展理念，紧扣我国社会主要矛盾变化，按照高质量发展的要求，统筹推进“五位一体”总体布局和协调推进“四个全面”战略布局，以供给侧结构性改革为主线，扎实做好稳增长、促改革、调结构、惠民生、防风险各项工作，推动质量变革、效率变革、动力变革，打好防范化解重大风险、精准脱贫、污染防治攻坚战，努力做到“五个一”，当好云南经济社会发展排头兵，全力以赴决战脱贫攻坚、决胜全面小康，奋力开创区域性国际中心城市建设新局面。

经济社会发展主要预期目标建议为：地区生产总值增长10%以上，一般公共预算收入增长5.5%，固定资产投资增长10%以上，社会消费品零售总额增长11%，城镇和农村常住居民人均可支配收入分别增长8%和8.5%，居民消费价格涨幅控制在3%以内，城镇登记失业率控制在4%以内。

围绕上述目标，我们将重点抓好九个方面的工作：

（一）着力培育新动能，夯实加快发展的产业支撑，促进经济发展提质增效

加快工业经济优化升级。深入实施工业强市战略，继续打好工业经济攻坚战，营造懂工业、谋工业、抓工业的浓厚氛围，确保规模以上工业增加值增长12%，新兴产业增加值占比提高到20%，工业和信息化投资增长10%以上。实施存量盘活工程。组织实施100项重点技改项目，重点抓好昆钢、中铝昆铜、云铝深加工等重大项目实施。强化结对帮扶包保责任制，实现全市规模以上企业和重点项目市县两级领导包保全覆盖。工业发展引导资金规模扩大到13亿元，组建市级再担保公司，继续推进财园助企贷融资，用好企业应急贷款周转金，着力破解企业融资难融资贵问题。加强政银企合作、银园合作，按季组织银企对接活动。加大电力市场化交易规模，确保降低企业用电成本20亿元以上。实施增量提升工程。建立开工、续建、竣工、引进“四个一批”重大项目库，开工建设紫光芯云等154个重点项目，续建东风云汽异地搬迁技改等84个重点项目，确保北方红外探测材料与器件产业化等69个重点项目竣工，完成年度投资365亿元以上，新增产值150亿元，力争汉能移动能源等20个项目落地开工。实施产业链培育工程。聚焦中高端关键链节，加快打造新能源汽车、轨道交通、石化、水电铝（硅）、稀贵金属新材料等8大产业链。实施中小企业成长工程。出台促进民营经济发展若干政策措施，重点扶持50户新兴产业类中小企业，全年新增规模以上工业企业不少于60户，其中新建投产20户。实施园区转型提升工程。建立多元化园区基础设施建设投资体系，确保全市园区完成基础设施投资120亿元。支持高新区开展容缺后补投资项目审批改革试点。建立完善项目入园、建设、投产、运营及退出机制，推动园区“腾笼换鸟”，确保创建3个以上省级10强园区。围绕规划实施、创新能力、产业集群、生态环境、节约集约用地、投资环境等方面，完善园区综合考核评价体系，引导园区走质量效益型发展道路。

推动服务经济提速。继续推进国家服务业综合改革试点，制定出台服务业倍增计划行动方案，大力推进500项在今后三年内滚动实施的服务业重大项目，确保服务业增加值增长10%以上。加快推进中国昆明国家大健康产业示范区建设和国家植物博物馆项目前期工作，开工建设鹏瑞利健康城等16个大健康项目。以巫家坝、会展中心片区和北京路、人民路沿线为重点，打造总部（楼宇）经济聚集区，新增总部企业8户以上，保有税收亿元以上楼宇20幢，培育十亿元楼宇1幢。加快昆明国际金融小镇示范点建设，加快昆明农村商业银行组建，金融机构人民币存贷款余额分别增长11%、12%。深入实施文化建设和产业发展“510”工程，力争文化产业增加值增长15%。落实“一部手机游云南”工作，加快晋宁未来城、七彩云南欢乐世界等重大项目建设，旅游业总收入突破2000亿元、增长30%，接待海外旅游者140万人次。推进华为软件开发云、中国数码昆明大数据产业园等项目建设，软件及信息技术服务业营业收入增长10%。发挥品牌展会示范效应，扶持特色专业会展品牌，会展业总收入突破30亿元、增长18%。加快空港物流聚集区、腾俊国际陆港等物流园区建设，创建3个省级示范物流园区。

大力培育发展新经济。积极推动以新技术、新产业、新业态、新模式为特征的新经济创新发展。加强创新平台建设，新增重点实验室和工程技术研究中心20个、院士工作站5个、县域科技成果转化中心8家，新增企业技术中心30家。重点围绕先进装备、生物医药、电子信息、新材料等领域，建设智能工厂、数字化车间，示范应用个性化定制、网络化协同制造、服务型制造等“新模式”，加快打造以经开区为核心的智能制造应用示范区。培育壮大本土电商品牌，大力引进知名电商企业，提升高新区国家电子商务示范基地发展水平，积极创建国家电子商务示范城市。制定促进分享经济发展的实施意见，清理制约分享经济发展的行政许可、商事登记等事项，加大政府部门对分享经济产品和服务的购买力度，推进分享经济健康快速发展。

（二）着力抓好城市规划建设，加强城市精细化管理，持续提升城市功能品质

强化规划龙头牵引作用。紧紧抓住昆明被国家列为城市总体规划和土地利用总体规划编制试点城市的重大机遇，举全市之力，用超常规举措，确保上半年上报新一轮城市总体规划成果，年内上报新一轮土地利用总体规划成果。加快推进“城市双修”工作，完成“五采区”生态修复和采区布点规划、二环路以内地区城市修补性规划。完成西南联大历史文化品牌相关规划、昆明城市米轨沿线改造提升规划，继续推进翠湖历史文化片区、文明街和南强街历史文化街区整治提升。完成昆明规划馆布展开馆，彰显昆明城乡规划新面貌。

持续改善交通基础设施。加快海口至易门、禄劝至会理（昆明段）高速公路前期工作，全力推进武倘寻、东格等13条高速公路建设，力争绕城高速东南段剩余段工程、功东高速主体工程完工，新增高速公路里程100公里以上。完成马郎立交节点工程建设。加快长水综合交通枢纽及东川、高新区通用机场前期工作，年内开工建设。开工新建城市道路38条，实施道路整治25条。开工建设滇缅大道快速路、三环闭合工程春雨路段和昌宏路段。完成人民路、东风路道路恢复提升工程，稳步推进南二环高架桥提升改造。实施金马坊节点改造工程，打通轨道及金碧广场地下空间。加快推进飞虎大道、春城路延长线、春雨路地下综合管廊建设。配合做好渝昆高铁建设。加快推进1号线西北延、2号线二期、4号线、5号线、6号线二期5条地铁在建项目。建设40个充电站、1400个充电桩。

改善城市人居环境。实施拆临拆违和旧城改造大会战，拆出昆明城市发展的新空间、拆出昆明市民居住的新环境，为主城产业发展“腾笼换鸟”创造条件。完成昆玉、东绕城等高速公路沿线，沪昆、云桂高铁沿线，以及13个重点片区共1500万平方米以上违法违规建筑治理拆除，实施主城区“三旧”改造连片开发600万平方米以上。实施创建全国文明城市三年行动计划，确保实现“六个不滑坡”“六个持续提升”，推动创建文明城市向建设城市文明跃升。做好迎接国家卫生城市复审、国家园林城市复查工作。加强智慧城市建设，重点推进智慧交通、决策支持平台、网格化管理、应急指挥等项目。采取“下泄、中疏、上截”防洪排涝措施，重点实施滇池海口闸出流能力提升“下泄”工程，城市河道及老旧排水管网改造等“中疏”工程，以及春雨路、西北三环、东大沟等城市面山洪水拦截“上截”工程，全面提升城市防洪排涝能力。深入实施交通综合整治提升工程，新建小花园空中走廊等11座人行天桥，加快推进机动车停车泊位扩容工程，新增机动车泊位1.2万个，进一步缓解城市交通拥堵。加快推进“公交都市”创建，新开公交线路10条。提升改造北京路、机场路城区段等城市道路绿化景观，新增城市绿地370公顷。

（三）着力实施乡村振兴战略，激发农业农村新活力，推动城乡融合发展

不断深化农村改革。建立健全农业转移人口市民化成本分担、财政转移支付动态调整、农业转移人口同城乡建设用地增减挂钩等机制。全面完成农村土地承包经营权确权登记颁证工作，抓好农村集体产权制度改革试点，推进承包地“三权分置”改革。完善农业支持保护制度，加大对结构调整、资源环境保护和科技研发等方面的支持力度。加快县区农村产权流转交易市场建设，建立全市四级农村产权流转交易服务体系。

加快建设现代农业。深化农业供给侧结构性改革，加快构建高原特色都市现代农业产业体系、生产体系、经营体系，争创国家农村一二三产业融合发展先导区，确保农业增加值增长5%。落实粮食安全行政首长责任制，稳定粮食生产。完成烤烟种植面积50万亩，争取烟农收入达到20亿元以上。积极推进蔬菜、花卉、山地牧业、林果、中药材等优势特色产业扩规模、调结构、上档次。大力发展休闲农业、乡村旅游等新产业新业态。发展壮大农产品加工产业集群，引进和培育达规农产品加工企业10个以上。开展农业质量提升行动，新增“三品一标”农产品40个。大力发展农村信息、技术服务，加快推进电子商务进农村工作。大力培育新型农业经营主体，新增省级龙头企业5个，新建农民专业合作社200个，培育新型职业农民2000人以上。

持续改善农村环境。实施公路安全生命防护工程，建设农村公路600公里。加快推进滇中引水骨干及配套工程建设，做好乌东德、白鹤滩水电站移民安置工作。续建4件中型、10件小型水库，新开工3件小型水库工程，实现柴石滩水库灌区工程石林片区通水。完成新一轮农村电网改造升级。加快推进燃气下乡工程，完成15个项目点火通气。扎实开展“七改三清”工作，完成乡镇生活污水处理设施建设任务，行政村生活垃圾有效治理率、公厕覆盖率达100%。全面落实村庄保洁制度，确保垃圾处理设施、保洁人员、管理制度“三到位”。

深入推进新型城镇化。科学制定全市乡村振兴战略规划。加快推进县（市）区新一轮城乡总体规划编制，稳步开展县域乡村建设规划编制，全面推行乡村建设规划许可证制度。出台支持晋宁融入主城发展的政策，加快主城六区一体化。推进嵩明、宜良撤县设市工作。着力加快斗南花卉、嘉丽泽国家一流特色小镇和九乡、轿子雪山、云上云、官渡古镇、凤龙湾阿拉丁省级一流特色小镇创建步伐。提高城镇化发展质量，加快农业转移人口市民化。

（四）着力深化改革，加快构建开放新格局，增强发展动力活力

推进改革增动力。深化供给侧结构性改革，稳妥推进“三去一降一补”，重点在“破”“立”“降”上下功夫。坚持去产能与优产能相结合，在转型升级中去落后产能，提高工业有效供给能力。按照中央和省的统一部署，统筹推进机构和行政体制改革。积极推进财税、国资国企等重点领域改革。深化“放管服”改革，加快建设政务一张网，全市行政审批业务系统全部接入政务服务网上平台，全面实现市县乡村四级行政审批和公共服务“一号申请、一窗受理、内部流转、一网通办”。公布市级第二批“最多跑一次”事项清单，推动“最多跑一次”改革向县（市）区延伸。在全市推行“3550”改革，对投资项目实行集中审批和全程免费代办服务。继续开展减证便民工作，凡没有法律法规依据的证明一律取消，能通过个人有效证照证明的一律取消，能采取申请人书面承诺方式解决的一律取消。

提升国际化水平。出台国际化建设工作方案。召开友城合作与发展高峰会，举办“海外昆明周”、外国使领馆国际推介活动，新增国际友城1个。加快铁路口岸规划申报，推进国家跨境电子商务综合试验区、市场采购贸易试点、文化出口基地创建工作，确保进出口总额增长16%以上。全力办好南博会、农博会等国际展会，举办国际性会议和展览62场次。加快教育、医疗国际化，力争在我市中小学就读的境外学生增长10%，2家医院通过国际医院联合体评审委员会认证。办好马拉松赛、龙舟赛、自行车赛等国际赛事。

提升招商引资成效。坚持把招商引资作为第一要事。开展大招商、招大商行动，市县两级领导要拿出主要精力抓招商。压实招商引资责任，层层签订招商引资目标责任书，做到目标明确、任务量化、责任落实。加强招商引资前期工作，研究出台全市年度招商引资实施方案，各县（市）区、各部门制定招商引资计划，充实完善招商引资项目库，包装一批重点产业招商项目，筛选提出一批拟招商企业目录，提升招商引资的针对性、实效性，在精准上下足功夫。全面落实招商项目推进领导“经理负责制”，切实提高签约率、落地率、开工率。确保引进市外到位资金1100亿元、增长10%以上，实际利用外资9亿美元、增长12%以上。

深入推进创新创造。积极配合创建“昆玉楚国家自主创新示范区”，建立金砖国家国际技术转移中心、昆明—万象科技创新中心，国际科技合作平台达70个。加大科技研发投入，力争全社会研发投入占GDP比重达2.3%以上。出台普惠性人才政策，对高校毕业生、留学归国人员和技能人才实行“零门槛”落户。实施海外高层次人才引进工程，引进境外智力项目14个。加快“全国质量强市示范城市”创建步伐，大力开展质量提升行动。采用国际和国外先进标准产品总量达40个，地方规范制定总量达17个，培育名牌产品60个以上。确保新增高新技术企业40家，高新技术企业营业收入增长12%以上，科技进步贡献率达55%。

深入推进市区融合发展。加快长水国际机场改扩建工程、机场至双龙高速等项目前期工作，实现机场北高速主线、呈黄快速、哨关大道等建成通车。合力推动先进装备制造、电子信息、生物医药、新材料、商贸服务产业集群发展，重点推进临空产业园、中关村·电子城科技产业园、空港商务区、智能终端产业园等产业发展平台建设，加快把滇中新区建成功能现代、产城一体、宜居宜业、融合发展的国际化高新产业新城。

（五）着力打好防范化解重大风险攻坚战，做好重点领域风险防范和处置，构建安全稳定的发展环境

防范和化解金融风险。尽快出台防范和化解金融风险攻坚战总体实施方案，以风险可控、金融服务实体经济、建立诚信健康金融环境为总体目标，以早识别、早预警、早处置为路径，加大风险排查力度，规范地方金融机构落地准入条件，建立省市共享综合监管信息平台，加大金融法规和金融知识的宣传普及教育。抓重点抓当前，对已暴露出的风险案件加快处置；抓识别强预警，对已显现有风险苗头的事项立即整改，避免形成风险；抓规范重监管，从源头上规避风险，营造诚信健康的金融发展环境，牢牢守住不发生区域性金融风险的底线。

防范和化解债务风险。尽快出台政府平台公司防风险实施方案，因时因势，调整完善公司融资条件，用足用好融资政策，清理各市级部门经营性资产，加快全市土地一级开发，通过注入优质资产和土地开发收益等各种有效举措，为各平台公司增信，确保债务链接，逐步降低负债率，做到能融资、融好资。研究出台化解政府债务风险实施方案，严格余额限额管理，摸清隐性债务底数，全面规范政府举债融资行为，确保政府债务可承受、可持续。要努力争取政府债券发行规模，多发、发好土地专债。强化风险预警控制，督促高风险地区集中资金、资源偿还存量债务，年末政府债务风险指标只降不升。严格偿债计划管理，压实主体责任，不断优化债务期限结构，防范到期风险。

防范和化解社会风险。认真贯彻总体国家安全观，深化反恐怖、反颠覆、反渗透、反分裂斗争，加强境外非政府组织服务管理，深化境外“三非”和宗教“三非”综合治理，确保全市社会政治稳定。以劳资、环保、征地拆迁、房地产等领域为重点，深入开展矛盾纠纷排查化解专项工作，做到发现在早、防范在先、处置在小。健全网络社会治理工作机制，构筑网络安全防线。

（六）着力打好精准脱贫攻坚战，确保脱贫质量，如期实现全面脱贫目标

围绕决战脱贫攻坚，聚焦“两不愁、三保障”总体目标，坚持精准扶贫、精准脱贫，保持财政投入的连续性，引导各方力量向贫困地区聚集，重点攻坚深度贫困，确保10万贫困人口脱贫，189个贫困村出列，贫困县区全部摘帽。

扎实推进精准施策。抓好重点县区农村危房改造工程，完成建档立卡户易地扶贫搬迁5593人。支持发展特色优势产业，建立资产收益扶持机制，实现户户有增收项目。加大订单、定向、定点培训，深入推进主城区转移就业帮扶，实现贫困户转移就业1.6万人。完善控辍保学动态归零督查机制，抓好贫困户成人教育，提高贫困户生产能力以及文化认知、社会交往能力。补齐健康扶贫短板，落实健康扶贫30条措施，落实家庭医生签约服务全覆盖，开展大病集中救治，完善基本医保、大病保险、医疗救助、兜底保障“四重”机制，落实贫困人口看病“一站式”服务，优化程序方便群众诊疗报结，确保符合分级诊疗、转诊转院规范的贫困人口看病医疗费用自付比例不超过10%。

提高精准帮扶水平。更加注重扶贫与扶志、扶智相结合，全面开展“双讲双评”“两学三比”活动，激发贫困地区干部群众内生动力。加强驻村工作队建设和驻村队员绩效管理，强化结对帮扶效果。着力推进市级单位和主城区、开发（度假）区对口帮扶贫困县区，实施“携手奔小康”行动，推动帮扶工作向产业发展、科教文卫等领域全方位延伸。鼓励企业、社会团体以项目合作、投资兴业、招工就业等多种形式积极参与扶贫。完善农村致富带头人帮扶机制，发挥脱贫攻坚农村致富带头人帮扶协会作用。

深入实施精准管理。扎实开展贫困对象动态管理和动态监测，做到“应识尽识，应纳尽纳，应扶尽扶，应退尽退”。落实扶贫专项资金公告公示制度，强化监察审计监管力度，开展扶贫领域专项整治。充分发挥“互联网+督查+脱贫攻坚”工作平台作用，实现脱贫攻坚工作督查全覆盖。

严格开展精准考核。紧扣关键指标，进行全方位自查评估，全力抓好问题整改“清零”。发挥考核“指挥棒”作用，严格落实脱贫攻坚责任制，切实做好贫困退出考核评价。

（七）着力打好污染防治攻坚战，狠抓滇池保护治理，建设生态良好美丽家园

全力推进以滇池为重点的水环境治理。实施滇池保护治理三年攻坚行动，强化落实河长制，全面推行湖（库）长制，突出抓好管网建设和雨季合流污染控制、主要入湖河道及支流沟渠治理、面源及内源污染治理、湿地生态环境效能提升等工作，加快推进草海及入湖河口清淤等43个重点项目，完成第十三水质净化厂建设等20个重点项目，实现35条入湖河道水质达标，草海水质达到IV类、外海水质保持Ⅴ类。持续开展阳宗海、牛栏江等重点流域水污染防治，加强松华坝、清水海、云龙水库等饮用水源地保护，确保全市水环境安全。

着力解决突出环境问题。持续抓好中央、省环保督查反馈问题整改，深入开展环保专项行动和环境安全隐患大排查。全面推行农业面源污染“一控两减三洁净”治理模式，加强以滇池流域为重点的农业面源污染防治。加大滇池流域及西山等重点区域采矿采砂采石点整治关停和“五采区”植被修复，确保年内完成全部修复任务30%以上修复面积。加强大气污染联防联控联治，强化建筑工地扬尘管理，在主城区全面推广使用智能渣土车，确保空气质量总体保持优良，达到国家二级标准，在全国的排位只能进位。

大力推动生态建设和绿色发展。确保安宁市创建成为省级生态文明县市。继续实施天保工程、退耕还林还草、石漠化综合治理等项目，实施营造林50万亩。推进县区海绵城市先行示范区建设，实施海绵城市建设20.8平方公里。推进垃圾分类和餐厨废弃物资源化利用。抓好冶金、化工、建材等重点行业节能降耗，确保完成省下达的万元GDP能耗下降和污染物减排任务。开展节约型机关、绿色家庭、绿色学校、绿色社区等创建行动。

（八）着力提高保障和改善民生水平，高质量办好惠民实事，增强人民群众获得感幸福感安全感

提高就业质量和收入水平。落实就业创业政策措施，继续推进“泛海扬帆昆明大学生创业行动”，深入实施微型企业培育工程，提高就业创业公共服务水平，确保实名登记高校毕业生就业率达90%以上，新增城镇就业12万人，农村劳动力转移就业15万人次。完善企业薪酬调查信息发布和工资指导线发布制度，开展人力资源市场秩序清理整顿、农民工工资支付保障等专项行动，全力维护劳动者合法权益。

提升社会保障服务水平。推进全民参保计划，确保各类社会保险参保覆盖率保持在96%以上，建档立卡贫困户家庭成员100%参加城乡居民基本医疗保险和大病保险，符合条件的100%参加城乡居民基本养老保险。加大社会救助力度，保障困难群众的基本生活。推进居家和社区养老服务改革试点、医养结合改革试点工作。加快建立多主体供给、多渠道保障、租购并举的住房制度，满足多层次住房需求。新开工8000套、建成2万套城镇保障性住房，缓解住房困难群体的住房问题。

提升社会事业发展水平。推动全域义务教育优质均衡。深入实施“三名”工程，引进名校8所，引进和培养名师44名、名校长8名。启动第三期学前教育行动计划，确保户籍儿童学前三年入园率保持在90%以上。推进城乡义务教育一体化发展，确保全市初中三年完学率达95%。启动实施高中阶段教育普及攻坚计划，高中阶段毛入学率达94%。力争昆明幼儿师范高等专科学校建成招生。实施健康昆明行动计划，加强基层医疗卫生服务体系和全科医生队伍建设，提高家庭医生签约服务水平和覆盖面，打造“15分钟社区健康服务圈”和“30分钟乡村健康服务圈”。引进北京天坛医院到昆合作办医。实施卫生科技人才培养“十百千”工程，开展国家干细胞临床项目研究。实施妇女儿童健康行动，开展出生缺陷产前基因检测。做好全国公共文化服务示范区创建工作，实施文化惠民、文艺精品、文化信息资源共享和数字图书馆推广工程，办好春城文化节等活动，加快聂耳大剧院、昆明美术馆等项目建设。实施文物保护利用项目15项以上，推进云南陆军讲武堂提升改造，启动文庙恢复性修建二期工程。实施全民健身计划，广泛开展群众性体育活动，提高竞技体育水平。

提高社会治理水平。推动社会治理重心向基层下移，完善社区网格化规范化治理机制，加强基层群众性自治组织规范化建设和社区基础服务设施建设，引导规范社会组织发展，加快打造共建共治共享的社会治理格局。坚持依法严惩、打早打小、除恶务尽，坚决打赢扫黑除恶专项斗争。落实安全生产责任，加大安全隐患排查整治力度，坚决遏制各类安全事故发生，积极创建国家安全发展示范城市。强化食品药品安全监管，深入推进国家食品安全示范城市创建。开展民族团结进步创建“六进”活动，推进“全国民族团结进步示范市”创建。加强国防教育、国防动员工作，完善双拥优抚安置体系，推动军民融合深度发展。

以造福人民为最大政绩，高质量办好“十件惠民实事”。

（九）着力加强政府自身建设，切实提高担当干事能力，努力打造人民满意的服务型政府

坚定政治方向。牢固树立“四个意识”，坚定“四个自信”，自觉用习近平新时代中国特色社会主义思想武装头脑、指导实践、推动工作，坚决维护以习近平同志为核心的党中央权威和集中统一领导，坚决贯彻中央方针政策，坚决落实省委、省政府和市委的决策部署，切实做到政令畅通，令行禁止。扎实开展“不忘初心，牢记使命”主题教育，引导广大干部永葆政治本色。

主动接受监督。自觉接受市人大及其常委会的法律监督和工作监督，严格执行市人大及其常委会决议决定，认真执行重大事项向市人大常委会报告制度。主动接受市政协和各民主党派、工商联、无党派人士的民主监督。切实做好人大常委会审议意见、人大代表建议和政协委员提案办理工作。进一步强化监察监督和审计监督。

严格依法行政。完善政府议事规则和决策程序，提高决策科学化、民主化、法治化水平。强化法治意识，坚持宪法和法律至上，认真执行宪法宣誓制度，落实党政主要负责人法治职责，深入推进“七五”普法和信用昆明建设，营造办事依法、遇事找法、解决问题用法、化解矛盾靠法的氛围。

持续改进作风。认真贯彻中央八项规定及实施细则精神，扎实开展形式主义和官僚主义专项整治，坚决防止“四风”问题反弹。大兴调查研究之风，练就“五个过硬”本领。建立“马上就办、真抓实干”工作推进机制，让“马上就办”成为各级干部的工作习惯，让“真抓实干”成为各级干部的自觉行动。

加强廉政建设。认真落实全面从严治党主体责任和“一岗双责”，深入推进政府系统党风廉政建设和反腐败斗争。坚持反腐败无禁区、全覆盖、零容忍，紧盯征地拆迁、扶贫攻坚、惠民利民等重点领域，聚焦侵犯群众切身利益的“微腐败”，持续推进廉洁政府建设，以实际行动树立政府良好形象。

各位代表！时代是出卷人，我们是答卷人，人民是阅卷人。新时代要有新气象，新征程要有新作为。让我们更加紧密地团结在以习近平同志为核心的党中央周围，在省委、省政府和市委的坚强领导下，深入学习贯彻党的十九大精神，不忘初心、牢记使命，锐意进取、务实苦干，为率先在全省全面建成小康社会，加快建设区域性国际中心城市而努力奋斗！
